# Acupuncture-related therapies for the poststroke cognitive impairment patients: A protocol for systematic review and network meta-analysis

**DOI:** 10.1097/MD.0000000000028735

**Published:** 2022-02-04

**Authors:** Jun Tian, Li-Bing Hu, Miao Wu, Wen-Li Mo, Peng Wu, Yao-Hui Pu, Han-Chao Yang, Yun Fan

**Affiliations:** a Hubei University of Chinese Medicine, Wuhan, Hubei, China; b Edong Healthcare Group, Huangshi Central Hospital, Huangshi, Hubei, China; c Hubei Provincial Hospital of Traditional Chinese Medicine, Wuhan, Hubei, China; d Hubei Province Academy of Traditional Chinese Medicine, Wuhan, Hubei, China.

**Keywords:** acupuncture, network meta-analysis, poststroke cognitive impairment

## Abstract

**Background:**

To compare and rank the clinical effects of different acupuncture and acupuncture-related therapies on patients with poststroke cognitive impairment.

**Methods:**

We evaluated the direct and indirect evidence from relevant studies using network meta-analysis. Eight databases were examined in order to find randomized controlled trials of acupuncture-related therapies for individuals with poststroke cognitive impairment. After 2 researchers independently scanned the literature, extracted the data, and assessed the risk of bias in the included studies, the data were analyzed using RevMan5.4, Stata15.0, and WinBUGS1.4.3 software.

**Results:**

We assess the benefits and drawbacks of various acupuncture-related therapies, rank the efficacy of various acupuncture-related therapies in the treatment of poststroke cognitive impairment, and describe the best acupuncture intervention approaches or combinations based on the available data.

**Conclusion:**

This study will contribute to the existence of data on the safety and efficacy of acupuncture-related therapies in the treatment of poststroke cognitive impairment, and it may aid clinical guideline makers in selecting the best acupuncture treatment for poststroke cognitive impairment.

**Registration Number:**

INPLASY2021120117.

## 1 Introduction

With the aging of the population is aggravating, the incidence of stroke in China is showing an increasing trend, and has become one of the main causes of disability and death in China.^[1]^ Patients with stroke are all at high risk of recurrence, which may lead to neurological deficits such as physical mobility, language ability, and cognitive function.^[2]^ Poststroke cognitive impairment (PSCI) is a relatively common poststroke complication, which can be divided into 2 stages in terms of disease severity: poststroke cognitive impairment no dementia (PSCIND) and poststroke dementia (PSD). PSCI not only affects the quality of life of patients after stroke, but also affects patients’ adherence to treatment, which in turn affects the survival time of patients. The China Stroke Report released in 2020 shows that the prevalence of stroke in China is 1114.8/100,000, with an annual incidence rate of 246.8/100,000 and a mortality rate of 149.49/100,000.^[3]^ Globally, China has become the country with the highest lifetime risk of stroke and disease burden.^[4]^ There is large heterogeneity in PSCI epidemiological data due to differences in study inclusion populations, missed visit rates, assessment tools, and diagnostic methods. Recently, large international cohort studies have reported that the prevalence of PSCI ranges from 24% to 53.4%,^[5,6]^ with the prevalence of PSD ranging from 11% to 42% and the prevalence of PSCIND ranging from 14% to 29%.^[7]^ Although neurological recovery is better in stroke patients under the age of 50 than in older patients, cognitive impairment still occurs in more than a large proportion of stroke patients under the age of 50.^[8]^ Without effective treatment, PSCIND patients will progress to PSD at an accelerated rate, and serious impact on quality of life and survival time. In addition, mortality was significantly higher in patients with PSCI than in those without cognitive impairment, with a 5-year survival rate of only 39% in patients with PSD compared with 75% in stroke patients of the same age without dementia.^[9]^ The high disability rate of PSCI reduces the quality of life, daily living ability, mental health status of patients, and significantly increases the burden of disease on families and society.^[10]^ Cholinesterase inhibitors and non-competitive N-methyl-D-aspartate receptor antagonists are currently the mainstays of many clinical treatment options, but their effects are limited and long-term use has certain side effects.^[11,12]^

In China, acupuncture, with unique efficacy, ease of operation, and few adverse effects, has a long history of application in the treatment of brain diseases. There is abundant evidence which shows that acupuncture has satisfactory efficacy in the treatment of vascular dementia, Parkinson disease, and depression. And it is used in the treatment of stroke,^[13]^ which can effectively improve symptoms such as hemiplegia of the limbs.^[14]^ The results of a recent network meta-analysis (NMA) show that, among many complementary alternative therapies, acupuncture may be the optimal and safest treatment for improving cognitive function in Alzheimer's disease patients.^[15]^ Whereas the inadequacy of pharmacological treatment, an increasing number of patients with PSCI are using non-pharmacological therapies, mainly acupuncture, as a complementary alternative therapy, confirming the potential of acupuncture in the treatment of stroke and cognitive impairment. However, due to the wide variety of acupuncture therapies, we still lack comparative studies between different acupuncture therapies. We propose to use the NMA method to evaluate the efficacy of multiple acupuncture methods in patients with PSCI, with the aim of providing evidence-based medical evidence for selecting the best acupuncture protocol for patients with PSCI.

## 2 Methods

This study has been registered with INPLASY, and registration number was INPLASY2021120117. This study will be reported according to the Preferred Reporting Items for Systematic Reviews and Meta-Analyses for Network Meta-Analysis Checklist (PRISMA-NMA)^[16]^ and the PRISMA for Acupuncture Checklist.^[17]^

### 2.1 Literature retrieval

Eight databases were searched in this study: PubMed, EMBASE, Cochrane Library, Web of Science, China Biology Medicine (CBM), the China National Knowledge Infrastructure (CNKI), Wanfang data, and the Chinese Scientific Journal Database (VIP). The search was conducted from the establishment of the database to December 1, 2021, with a combination of MeSH terms and free words (Table 1).

**Table 1:** Detailed search strategy for PubMed.

No.	Search item
#1	Acupuncture[MeSH Terms]
#2	Acupuncture Points[MeSH Terms]
#3	Acupuncture, Ear[MeSH Terms]
#4	Acupuncture Analgesia[MeSH Terms]
#5	Acupuncture Therapy[MeSH Terms]
#6	Auriculotherapy[MeSH Terms]
#7	Acupuncture^∗^[Title/Abstract] OR Needling[Title/Abstract] OR Electroacupuncture^∗^ [Title/Abstract] OR Electro-acupuncture [Title/Abstract] OR Acupoint Therapy[Title/Abstract] OR Acupuncture Treatment[Title/Abstract] OR Acupuncture Treatments[Title/Abstract] OR Needle Therapy[Title/Abstract] OR silver needle[Title/Abstract] OR moxibustion [Title/Abstract] OR de qi[Title/Abstract] OR meridian [Title/Abstract] OR Auriculotherapy[Title/Abstract] OR needle pricking[Title/Abstract] OR Transcutaneous Electric Nerve Stimulation [Title/Abstract] OR acupressure[Title/Abstract] OR needling[Title/Abstract] OR intradermal needle[Title/Abstract] OR Point application[Title/Abstract] OR fire needle[Title/Abstract] OR fire needling[Title/Abstract] OR three-edged needle[Title/Abstract] OR blood letting Therapy [Title/Abstract] OR pricking blood therapy[Title/Abstract] OR a-shi point [Title/Abstract] OR point injection [Title/Abstract] OR Hydro acupuncture[Title/Abstract] OR Needle Warming Therapy[Title/Abstract] OR scalp acupuncture[Title/Abstract] OR auricular acupuncture[Title/Abstract] OR ear acupuncture[Title/Abstract] OR intradermal needling[Title/Abstract]
#8	#1 OR #2 OR #3 OR #4 OR #5 OR #6 OR #7
#9	stroke[MeSH Terms]
#10	brain ischemia[MeSH Terms]
#11	post stroke[Title/Abstract] OR post-stroke[Title/Abstract] OR post-stroke^∗^[Title/Abstract] OR stroke[Title/Abstract] OR brain ischemia[Title/Abstract]
#12	#10 OR #11
#13	dementia[MeSH Terms]
#14	memory disorders[MeSH Terms]
#15	neurocognitive disorders[MeSH Terms]
#16	neurocognitive disorders/[MeSH Terms]
#17	cognitive dysfunction[MeSH Terms]
#18	cognition disorders[MeSH Terms]
#19	cognition impairment[MeSH Terms]
#20	dementia[Title/Abstract] OR memory disorders[Title/Abstract] OR neurocognitive disorders[Title/Abstract] OR neurocognitive disorders[Title/Abstract] OR cognitive dysfunction[Title/Abstract] OR cognition disorders[Title/Abstract] OR cognition impairment[Title/Abstract]
#21	#13 OR #14 OR #15 OR #16 OR #17 OR #18 OR #19 OR #20
#22	Randomized Controlled Trial [Publication Type]
#23	Randomized Controlled Trials as Topic[MeSH Terms]
#24	Randomized Controlled Trial[All Fields] OR random^∗^[Title/Abstract] OR RCT[All Fields] OR Trial^∗^[All Fields]
#25	#22 OR #23 OR #24
#26	#8 AND #12 AND #21 AND #25

### 2.2 Inclusion criteria

Published clinical randomized controlled trials of acupuncture-related therapies for PSCI.

Patients were required to have survived the acute phase and have clear diagnostic criteria: ① Definitive stroke diagnosis; ② The presence of cognitive impairment; ③ Cognitive impairment that appears after the stroke event and persists until 3 to 6 months.

We chose various types of acupuncture-related therapies as the treatment groups, including simple acupuncture, electroacupuncture, warm acupuncture, auricular acupuncture, or a combination of acupuncture and medication. The control group was a comparison between various types of western drugs that improve cognitive function (such as donepezil hydrochloride, nimodipine, etc) or various types of acupuncture-related therapies. At least one of the following outcome indicators was required to be reported: Effectiveness Rate, Mini-Mental State Examination, and Alzheimer Disease Assessment Scale-Cognitive section.

### 2.3 Exclusion criteria

① Non-randomized controlled trials; ② Studies without clear diagnostic criteria; ③ Review, case report, or meta-analysis; ④ Studies lacking clear primary data; ⑤ Interventions did not include studies of various forms of acupuncture; ⑥ Repeatedly reported studies.

### 2.4 Data extraction

Two researchers individually finished the literature screening, data extraction, and finally cross-checked. The third researcher would make the final decision if there was any disagreement. In case of disagreement, the decision was referred to the third researcher. A uniform data extraction form was used, containing the authors’ names, journal name, year of publication, sample size of each group, age of subjects, type of intervention, frequency of treatment, treatment period pre- and posttreatment data, randomization method, allocation concealment, and blinding, etc.

### 2.5 Risk assessment of bias

Two researchers assessed the risk of bias of the included studies in accordance with the risk of bias assessment tool recommended in Cochrane Handbook 5.1.^[18]^

### 2.6 Statistical analysis

RevMan 5.4 (Cochrane Collaboration, Oxford, UK), Stata 15.0 (Stata Corporation, College Station, Texas), and WinBUGS 1.4.3 (MRC Biostatistics Unit, Cambridge, UK) software were used performing the statistical analysis.^[19–21]^

The statistical data were analyzed using the odds ratio as the efficacy statistic; the measurement data were expressed as the weighted mean difference or standardized mean difference. All effect sizes are expressed as 95% confidence intervals.

(1) After risk of bias evaluation by 2 researchers, risk of bias evaluation plots were performed using RevMan 5.4 software.
(2) Using Stata 15.0 to draw a network diagram to determine direct and indirect comparison relationships between various interventions.^[22]^
(3) Traditional pair-wise meta-analysis was performed using Stata 15.0 to evaluate the clinical effects of the 2 treatments in all original studies with direct comparisons
(4) NMA was performed by using WinBUGS 1.4.3; node-plit model was used for inconsistency test, and if there was no statistical difference (*P* > .05), consistency model was used for analysis. Conversely, inconsistency model was used.
(5) We used the Stata 15.0 to draw the funnel plots, determining whether there was evidence of a small sample effect in the included research.^[23]^
(6) The surface under the cumulative ranking curve were generated by using Stata 15.0, with higher surface under the cumulative ranking curve scores implying higher treatment grades.^[24]^

## 3 Discussion

As the world population ages, the incidence of stroke continues to rise and is becoming younger. National stroke screening data show that the standardized incidence of first stroke in people aged 40 to 74 rose from 189/100,000 in 2002 to 379/100,000 in 2013, an average annual increase of 8.3%.^[25,26]^ Stroke is characterized by high incidence, disability, mortality, and recurrence rates. 2016 Global Burden of Disease data show that stroke is the first cause of year of death in China.^[4]^ Globally, 90.7% of stroke is associated with correctable risk factors such as hypertension, diabetes, dyslipidemia, heart disease, smoking, alcohol intake, unhealthy diet, and abdominal obesity.^[27,28]^ Once cognitive impairment occurs, it will further affect the patient's degree of cooperation with treatment, thus affecting the treatment of stroke itself, so timely and effective improvement of cognitive function is particularly important for stroke recovery. Current approaches to improving cognitive function mostly follow protocols for mild cognitive impairment. Although impaired cognitive function will gradually recover in some patients as their stroke remission, 11% to 42% of patients will experience progressive cognitive deterioration and eventually develop to PSD.^[7]^ Once a patient is diagnosed with PSD, the burden on the family and society will be enormous.^[29,30]^

Acupuncture, as a characteristic therapy of traditional Chinese medicine, is clinically effective in a variety of diseases. Modern research and clinical outcomes have confirmed the role of acupuncture in stroke and cognitive impairment.^[31]^ It can be used as a safe and effective complementary alternative therapy for the treatment of PSCI,^[32]^ considering it is safe and no side effect. In addition, a good doctor-patient interaction during acupuncture is beneficial in restoring patient confidence in treatment, improving symptoms, reducing tension, and building a trusting doctor-patient relationship.

Based on the available evidence, we synthesized and compared the advantages and disadvantages of various acupuncture-related therapies, ranked the efficacy of various acupuncture-related therapies for the primary treatment of PSCI, and summarized the best acupuncture intervention or combination. This study will evaluate the safety and efficacy of various acupuncture-related therapies for the treatment of PSCI and provide new clinical evidence as complementary alternative therapies for the treatment of PSCI, which may help guide the clinical selection of more appropriate interventions.

## Author contributions

**Data curation:** Jun Tian, Li-Bing Hu.

**Formal analysis:** Wen-Li Mo.

**Funding acquisition:** Yun Fan.

**Methodology:** Miao Wu, Yao-Hui Pu.

**Resources:** Peng Wu, Han-Chao Yang.

**Software:** Peng Wu, Yao-Hui Pu.

**Supervision:** Jun Tian, Li-Bing Hu, Yun Fan.

**Validation:** Miao Wu, Wen-Li Mo.

**Writing – original draft:** Jun Tian, Li-Bing Hu.

**Writing – review & editing:** Han-Chao Yang, Yun Fan.
